# Global trends in resistance studies of gemcitabine and pancreatic cancer: a bibliometric and visual analysis from 2010 to 2024

**DOI:** 10.3389/fphar.2025.1564561

**Published:** 2025-04-25

**Authors:** Dandan Gu, Shaoyang Huang, Kai Zhao, Xiaohong Zhang, Jinjing Zhang, Wei Xiong

**Affiliations:** ^1^ Department of Gastroenterology, Northeast Yunnan Regional Central Hospital, Zhaotong, China; ^2^ College of Life Sciences, Shaanxi Normal University, Xi’an, China; ^3^ Department of Biochemistry and Molecular Biology, School of Basic Medical Sciences, Dali University, Dali, China; ^4^ Key Laboratory of Clinical Biochemistry Testing in Universities of Yunnan Province, School of Basic Medical Sciences, Dali University, Dali, China

**Keywords:** pancreatic cancer, gemcitabine, drug resistance, citespace, VOSviewer, bibliometric analysis

## Abstract

**Introduction:**

Pancreatic adenocarcinoma (PC) represents a prevalent and highly aggressive malignancy within the digestive system, characterized by an exceedingly poor prognosis and a dismal 5-year survival rate of below 7%. Gemcitabine (GEM) remains the cornerstone chemotherapeutic agent in the management of PC; however, the growing challenge of GEM chemoresistance, which undermines treatment efficacy, represents a significant obstacle in clinical practice. To date, no comprehensive bibliometric analysis has been undertaken to systematically explore studies on GEM resistance in the context of PC. This study aims to deliver a thorough evaluation of the research hotspots pertaining to GEM resistance in PCs.

**Method:**

A systematic search was conducted for articles published from 1 January 2010, to 15 December 2024, focusing on resistance studies of GEM in PC. Bibliometric analysis and visualization were performed utilizing VOSviewer and CiteSpace tools, applied to literature data extracted from the Web of Science Core Collection (WoSCC).

**Results:**

Between 2010 and 2024, a total of 2,689 papers were published across 472 institutions in 74 countries, reflecting a consistent upward trajectory in annual publication output. China and Fudan University emerged as the leading contributors to the research output on this topic, representing the most prolific country and institution, respectively. Giovannetti, Elisa, and Yu, Xianjun are the most prolific scholars in this field. *Cancer Research* stands out as the most cited and impactful journal, while research on the tumor microenvironment, targeted therapy, and circular RNA has emerged as a key focus area in recent years.

**Conclusion:**

This study provides a systematic and comprehensive overview of the literature on GEM resistance in PC over the past 15 years. This analysis offers scholars critical insights into the field from a bibliometric perspective, potentially informing future studies on the development of chemotherapeutic treatments for PC.

## 1 Introduction

Pancreatic cancer (PC), renowned for its aggressive nature within the digestive system, is associated with a dismal prognosis, as its global incidence and mortality rates have been steadily increasing in recent years. Although PC accounts for only 2%–3% of all cancer cases, it is responsible for a disproportionately high mortality rate of 7%, ranking it as the third leading cause of cancer-related deaths, surpassed only by lung and colorectal cancers (Siegel et al., 2024). By 2030, PC is projected to surpass other malignancies and become the second leading cause of cancer-related deaths worldwide, following lung cancer (Rahib et al., 2014). The asymptomatic nature of early-stage PC results in the majority of patients being diagnosed with advanced or metastatic disease, leaving only 10%–15% of newly diagnosed cases eligible for surgical resection. Furthermore, many patients experience local recurrence or distant metastasis within one to two years post-surgery, significantly compromising long-term survival outcomes (Karunakaran and Barreto, 2021; Schneider et al., 2021). Consequently, the majority of patients rely on non-surgical interventions, including conventional chemotherapy, radiotherapy, and targeted molecular therapies. Despite advancements in surgical techniques and chemotherapeutic regimens, the five-year survival rate for PC remains alarmingly low, at less than 9% (Conroy et al., 2023). Systemic therapy has emerged as a cornerstone strategy for extending patient survival in oncology. Despite the substantial prognostic improvements achieved with FOLFIRINOX and GEM-based chemotherapy regimens (Marschner et al., 2023), their therapeutic efficacy remains constrained by inherent biological barriers, including tumor microenvironment-induced immunosuppression, extensive mutational complexity (e.g., high-frequency mutations in KRAS and TP53), and acquired chemoresistance (Allen et al., 2023; Mondal et al., 2023). Contemporary systemic therapeutic approaches for pancreatic cancer aim to transcend conventional treatment paradigms, incorporating molecularly stratified targeted therapies (e.g., KRAS G12C inhibitors) (Keane et al., 2024), immune-combination strategies (e.g., PD-1/CTLA-4 dual-antibody blockade in synergy with chemotherapy) (Mishra et al., 2024), and advanced drug delivery platforms (e.g., nanocarriers and antibody-drug conjugates) designed to potentiate therapeutic efficacy (Chen Y. et al., 2023; Tsimberidou et al., 2023). Furthermore, liquid biopsy-enabled dynamic monitoring and personalized dosing strategies (Sekita-Hatakeyama et al., 2022) offer novel avenues to mitigate tumor heterogeneity and combat the evolution of drug resistance.

Chemotherapy remains a cornerstone of multimodal PC treatment, with GEM established as the first-line chemotherapeutic agent for patients with PC, particularly those unsuitable for surgical intervention (Beutel and Halbrook, 2023). Notably, GEM, a pyrimidine analog, relies on specific nucleoside transporter proteins (NTs) to efficiently traverse the plasma membrane. Its mechanism involves replacing cytosine during DNA replication and inhibiting deoxyribonucleotide biosynthesis, thereby suppressing PC cell proliferation (Binenbaum et al., 2015). Resistance to GEM may manifest as either intrinsic or acquired following repeated treatment cycles. Insights from sophisticated fields like proteomics and advanced RNA sequencing reveal that various proteins play a part in the resistance to GEM. Moreover, most patients either develop or swiftly develop resistance to GEM, greatly reducing its effectiveness and worsening the adverse survival rates in PC patients (Jain and Bhardwaj, 2021; Zeng et al., 2019). Consequently, elucidating the mechanisms of GEM resistance and identifying novel therapeutic targets have emerged as pivotal challenges in contemporary biological and clinical research.

Despite a plethora of studies on GEM resistance in PC, the overarching development trajectory, current landscape, and emerging research hotspots in this domain remain inadequately characterized. This research marked the first use of bibliometric analysis to methodically amalgamate pertinent studies spanning 15 years, utilizing the Web of Science Core Collection (WOSCC) database in conjunction with CiteSpace and VOSviewer, aiming to thoroughly pinpoint and examine research focal points and ultimately deliver solid, data-oriented perspectives for experts in this domain.

## 2 Materials and methods

### 2.1 Data sources and search strategy

To identify relevant articles, we conducted a comprehensive search in the Web of Science Core Collection (WoSCC) for publications released between 1 January 2010, and 15 December 2024. To maintain consistency and eliminate potential temporal biases in database entries, the search process was meticulously completed within a single day. The search utilized the formula: TS = ((Gemcitabine) AND ((chemoresistance OR chemotherapy resistance* OR chemotherapy drug resistance* OR chemotherapy-refractory drug* OR chemotherapeutic drug* OR drug resistance*)) AND (“pancreatic cancer” OR “pancreatic carcinoma” OR “pancreatic neoplasm” OR “cancer of pancreas” OR “carcinoma of pancreas” OR “neoplasm of pancreas” OR “pancreatic ductal adenocarcinoma” OR PDAC)) AND DOP = (2010-01-01/2024-12-15).

**FIGURE 1:**
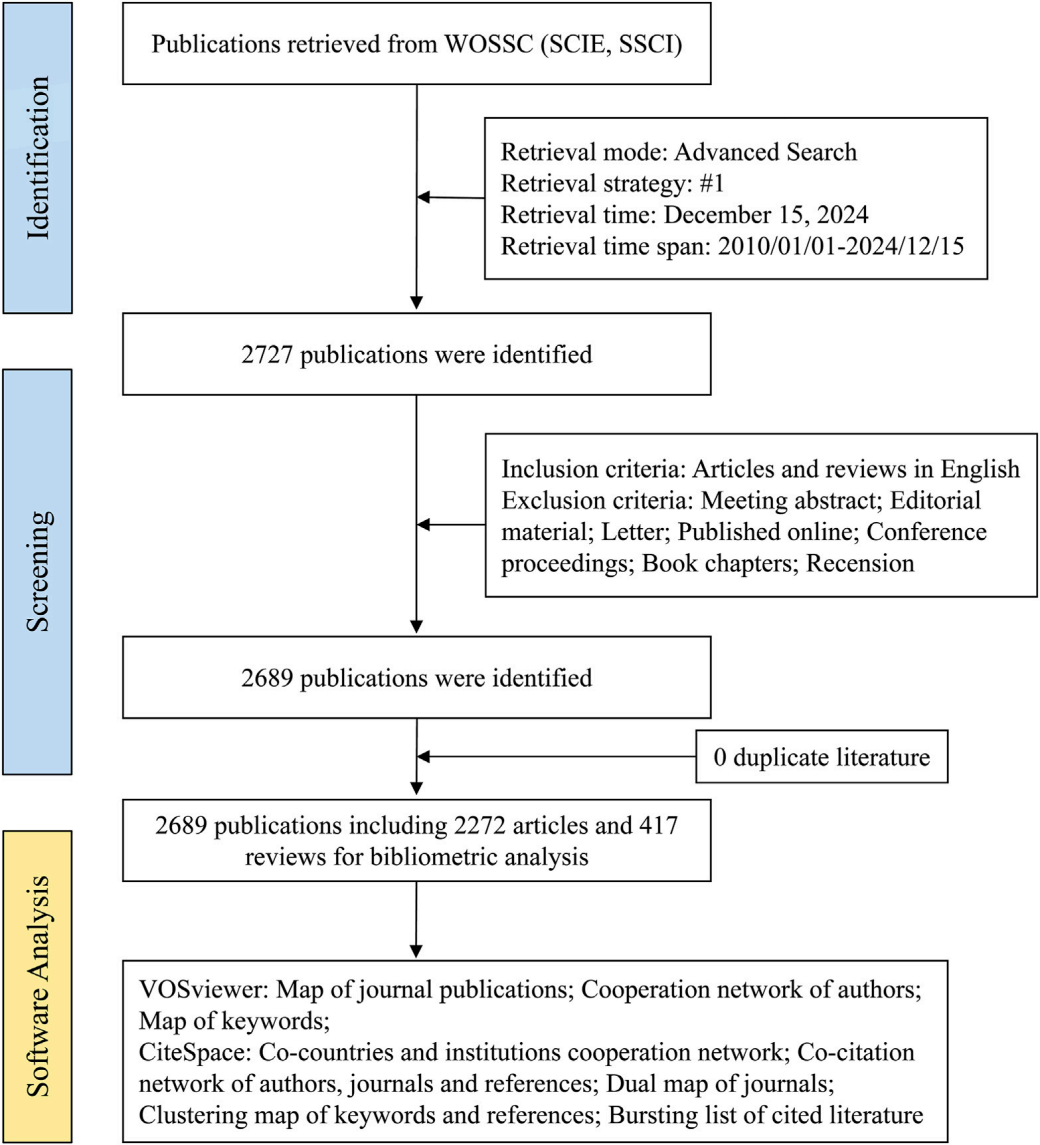
The flowchart of this study.

The literature search was restricted exclusively to peer-reviewed journal articles. To enhance the precision and reliability of our bibliometric analysis, each retrieved article underwent a rigorous evaluation to ascertain its adherence to the inclusion criteria, namely: (1) direct relevance to the study of GEM resistance in PC, and (2) publication in the English language. Articles were excluded if they met the following criteria: (1) lack of relevance to the investigation of GEM resistance in PC, or (2) classification as a review article, conference abstract, case report, letter, or preprint. The selected articles were subsequently exported in plain text format for further analysis. Ultimately, a total of 2,689 publications pertaining to GEM resistance in PC were identified and included in the study (Figure 1).

### 2.2 Data analysis

The aforementioned literature data were exported in TXT format, followed by analysis and visualization using advanced bibliometric tools such as VOSviewer (v1.6.18), CiteSpace (v6.1. R2 Basic), and Microsoft Excel 2010. CiteSpace, an advanced software developed by Dr. Chaomei Chen in the United States, facilitates the visualization and in-depth analysis of scientific literature. CiteSpace, developed by Dr. Chaomei Chen in the United States, serves as a robust platform for visualizing and analyzing scientific literature, enabling researchers to examine citation networks, author collaborations, and topic evolution comprehensively. This tool provides a deeper understanding of emerging trends and research hotspots in the field (Chen, 2004). It is mainly used in this study to generate visual network maps of countries and institutions, authors, journals, and reference outbreaks (Chen H. et al., 2023).

The VOSviewer tool, an advanced bibliometric tool, is extensively used to chart the evolving and structural elements of scientific understanding. It enables various analytical methods like co-word, co-citation, document coupling, and detailed visualization. In this research, VOSviewer was utilized to create visual representations of nations, writers, joint institutions, referenced journals, and keyword correlations, as well as to develop density maps (Ma et al., 2021). The primary objective of VOSviewer lies in providing users with an in-depth understanding of the intricate dynamics and overarching structure of scientific research.

## 3 Result

### 3.1 Annual publication volume and trend analysis

**FIGURE 2:**
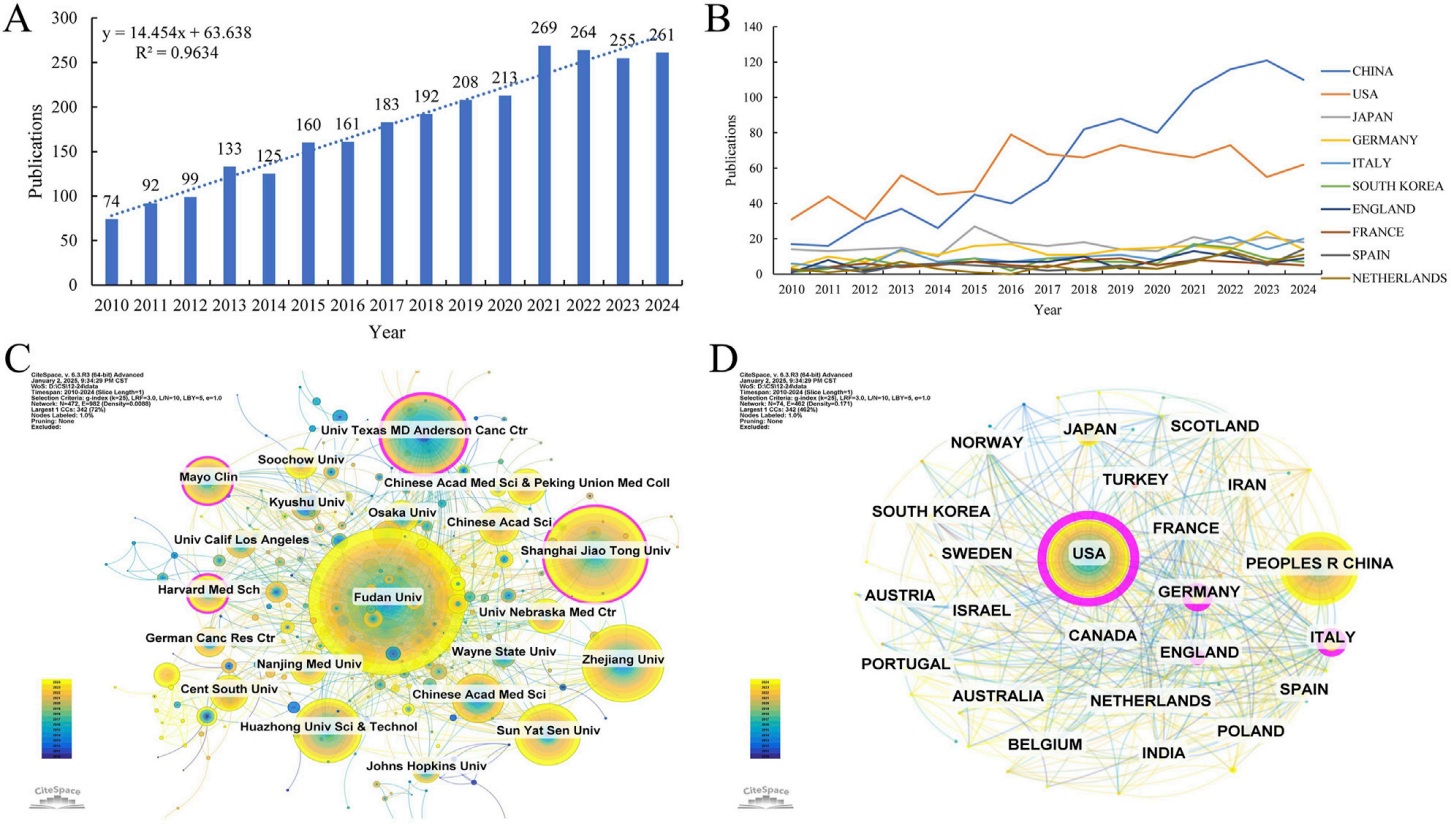
**(A)** Annual volume of publications. **(B)** Line graph of national publications. **(C)** Networks of country cooperation. **(D)** Networks of institutional co-operation.

This study incorporated 2,689 papers from the WOS core database, with the annual publication trends on GEM and PC resistance from 2010 to 2024 depicted in (Figure 2A). Over the past decade, international research outputs on GEM resistance in PC have demonstrated a significant and steady upward trajectory. Research output reached a peak of 269 articles in 2021, with publication numbers remaining consistently high thereafter, underscoring the growing scholarly focus on GEM resistance in PC treatment.

### 3.2 Analysis of country/institution publications and collaborations

Studies on GEM resistance in PC have been conducted by 74 countries and institutions worldwide. Figure 2B demonstrates the yearly publication patterns across the last ten nations. Concurrently, we’ve tallied countries with the greatest volume of publications and citations (Table 1). In terms of publishing volumes, China, the United States, Japan, Germany, and Italy stand out as the five nations with the highest outputs, with respective outputs of 964, 865, 249, 197, and 160. Notably, China and the United States contributed 35.85% and 32.17% of the total publications, respectively, significantly surpassing Japan (9.26%), Germany (7.33%), and Italy (5.95%). It is noteworthy that China has demonstrated a markedly rapid increase in research output in this domain over the past decade. Regarding citation impact, publications from the United States exhibit the highest citation count (40,774) and centrality score (0.52). Moreover, the citation-to-publication ratio stands at 47.14, substantially exceeding that of other nations, underscoring the prominence and high quality of U.S.-based research in this field. Publications from China have garnered a total of 26,340 citations, with an average citation per paper of 27.32, trailing behind Germany (39.84), Italy (32.54), and Japan (28.80). This disparity suggests that Chinese researchers should enhance the visibility and academic impact of their contributions in this domain. With respect to international collaboration, the United States occupies a central position within the global research network, fostering extensive partnerships with other nations. Collaborative efforts among the United States, China, France, the United Kingdom, Germany, Sweden, and Canada have resulted in a higher frequency of joint publications and a greater volume of research output.

**TABLE 1:** Statistics of top countries in terms of number of publications (top 10).

Rank	Country	Publications (2,689, %)	Centrality	Citations	Citation per publication
1	China	964 (35.85%)	0.1	26,340	27.32
2	United States	865 (32.17%)	0.52	40,774	47.14
3	Japan	249 (9.26%)	0.06	7,170	28.80
4	Germany	197 (7.33%)	0.21	7,849	39.84
5	Italy	160 (5.95%)	0.12	5,207	32.54
6	South Korea	112 (4.17%)	0.05	2,681	23.94
7	England	102 (3.79%)	0.1	5,956	58.39
8	France	85 (3.16%)	0.07	3,577	42.08
9	Spain	79 (2.94%)	0.08	2,672	33.82
10	Netherlands	70 (2.60%)	0.01	2,491	35.59

Between 2010 and 2024, a total of 472 institutions worldwide have published research papers addressing the issue of GEM resistance in PC. Using CiteSpace software to designate institutions as nodes, we constructed a comprehensive international institutional collaboration network (Figure 2C). Additionally, we methodically measured the quantity of published papers and their citation rates (Figure 2D; Table 2). Concerning the volume and impact of publications, key institutions such as Fudan University, Shanghai Jiao Tong University, the University of Texas MD Anderson Cancer Center, Zhejiang University, and Huazhong University of Science and Technology, predominantly situated in China, are well-known. The University of Texas MD Anderson Cancer Center is prominent for having the most citations for published papers (n = 6,754) and the highest average number of citations per article (n = 100.81), highlighting its significant impact and acknowledgment in the scholarly realm. The analysis of institutional collaboration networks reveals that Fudan University occupies a central position, demonstrating strong international connectivity and fostering robust partnerships with other global institutions.

**TABLE 2:** Statistics on top institutions with the highest number of publications (top 10).

Rank	Institution	Publications (2,689, %)	Citations	Citation per publication
1	Fudan Univ	114 (4.24%)	3,510	30.79
2	Shanghai Jiao Tong Univ	77 (2.86%)	2,652	34.44
3	Univ Texas Md Anderson Canc Ctr	67 (2.49%)	6,754	100.81
4	Zhejiang Univ	62 (2.31%)	1,448	23.35
5	Huazhong Univ Sci & Technol	52 (1.93%)	1981	38.10
6	Sun Yat Sen Univ	51 (1.90%)	1,507	29.55
7	Chinese Acad Med Sci	41 (1.52%)	1892	46.15
8	Mayo Clin	38 (1.41%)	1,074	28.26
9	Chinese Acad Sci	34 (1.26%)	1,064	31.29
10	Univ Nebraska Med Ctr	33 (1.23%)	2,159	65.42

### 3.3 Analysis of journals and cited journals

**FIGURE 3:**
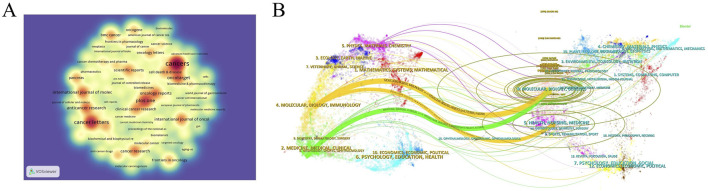
**(A)** Density map of journal publications. **(B)** Dual map of journals: the colored tracks represent citation connections, with citing journals on the left and cited journals on the right.

Using the journal function of VOSviewer software, we constructed a density plot to visually illustrate the distribution of journal publishing studies on GEM resistance in PCs. Regarding journal publications (Table 3), the density plot (Figure 3A) highlights *Cancers* (n = 120, IF = 4.5, Q1), *Cancer Letters* (n = 81, IF = 9.1, Q1), and *PLOS ONE* (n = 64, IF = 2.9, Q1) as the top three journals. These journals are pivotal publication venues warranting close attention from scholars in this field. In terms of citations, *Cancer Research* (n = 414, IF = 12.5, Q1), *Clinical Cancer Research* (n = 1768, IF = 10.4, Q1), and *Journal of Clinical Oncology* (n = 1,434, IF = 42.1, Q1) emerge as the top three journals. Their consistently high impact factors underscore their significant influence within the research domain. Additionally, the predominantly influential status of these journals reinforces their significant role in examining GEM resistance in PC (Table 4). The leading 10 highly frequently referenced journals achieve a Q1 ranking, with their publications greatly propelling the research into GEM resistance in PC. Additionally, we overlaid citing and cited journals to visualize their disciplinary relationships. Journals cited were arranged on the left side, while those cited were on the right side in the graph (Figure 3B). The analysis revealed two prominent colored citation pathways reflecting distinct research domains: studies originating in “Molecular/Biology/Immunology” journals frequently appear in “Molecular/Biology/Genetics” and “Health/Nursing/Medicine” journals, while literature from “Medicine/Medical/Clinical” journals is predominantly cited by articles within the same category.

**TABLE 3:** Statistics on top journals with the highest number of publications (top 10).

Rank	Journal	Publications (2,689, %)	Impact factor	Quartile in category
1	Cancers	120 (4.46%)	4.5	Q1
2	Cancer Letters	81 (3.01%)	9.1	Q1
3	PLOS ONE	64 (2.38%)	2.9	Q1
4	Oncotarget	51 (1.90%)	-	-
5	International Journal of Molecular Sciences	48 (1.79%)	4.9	Q1
6	Anticancer Research	47 (1.75%)	1.6	Q3
7	Cancer Research	43 (1.60%)	12.5	Q1
8	Oncology Reports	43 (1.60%)	3.8	Q1
9	International Journal of Oncology	42 (1.56%)	4.5	Q1
10	Scientific Reports	37 (1.38%)	3.8	Q1

**TABLE 4:** Statistics of most cited journals (top 10).

Rank	Journal	Co-citations	Impact factor	Quartile in category
1	Cancer Research	2,152	12.5	Q1
2	Clinical Cancer Research	1,768	10.4	Q1
3	Journal of Clinical Oncology	1,434	42.1	Q1
4	Oncogene	1,327	6.9	Q1
5	CA-A Cancer Journal for Clinicians	1,297	521.6	Q1
6	Nature	1,255	50.5	Q1
7	New England Journal of Medicine	1,254	96.3	Q1
8	British Journal of Cancer	1,237	6.4	Q1
9	PLOS ONE	1,175	2.9	Q1
10	Proceedings Of The National Academy Of Sciences Of The United States	1,131	9.4	Q1

### 3.4 Analysis of authors and cited authors

**FIGURE 4:**
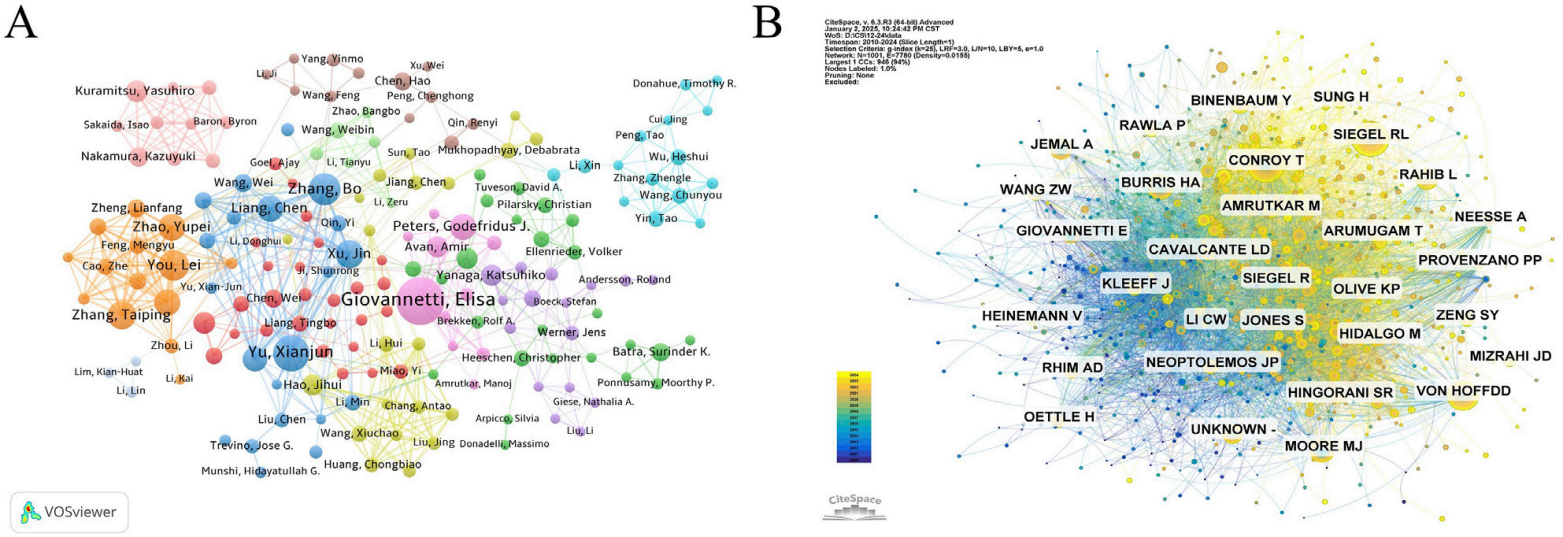
**(A)** Cooperation network of authors. **(B)** Co-citation network of authors.

Researchers delving into GEM resistance in PC exhibit the highest publication and citation numbers in (Table 5). In the diagram depicting author collaboration (Figure 4A), the node size signifies the number of times authors appear, and the connecting lines indicate their collaboration. Thicker lines indicate stronger collaborative relationships among authors. Giovannetti, Elisa (n = 38), Yu, Xianjun (n = 27), and Zhang, Bo (n = 23) are the leading contributors, as evidenced by their significant number of publications in this research domain. Regarding author collaboration, Giovannetti, Elisa serves as a pivotal figure within the research network, characterized by extensive scholarly connections. Additionally, Chinese scholars, including Yu, Xianjun, Zhang, Bo, Liang, Chen, and Xu, Jin, demonstrate close interactions, collaboratively generating a significant body of research. Tuveson, David A. (n = 2,445), Hidalgo, Manuel (n = 1814), and Giovannetti, Elisa (n = 1,601) rank as the most influential authors in this field, based on their high citation counts. Indicating that the work of these scholars has profoundly influenced the field of GEM resistance research in PC (Figure 4B).

**TABLE 5:** Top authors in terms of publications and citations (top 10).

Rank	Label	Documents	Rank	Label	Citations
1	Giovannetti, Elisa	38	1	Tuveson, David A.	2,445
2	Yu, Xianjun	27	2	Hidalgo, Manuel	1,814
3	Zhang, Bo	23	3	Giovannetti, Elisa	1,601
4	You, Lei	22	4	Jodrell, Duncan I	1,446
5	Xu, Jin	20	5	Neesse, Albrecht	1,405
6	Zhang, Taiping	20	6	Zhao, Yupei	1,269
7	Zhao, Yupei	19	7	You, Lei	1,268
8	Liang, Chen	18	8	Maitra, Anirban	1,239
9	Peters, Godefridus J	18	9	Peters, Godefridus J	1,212
10	Yang, Gang	18	10	Yu, Xianjun	1,157

### 3.5 Keyword network analysis

**FIGURE 5:**
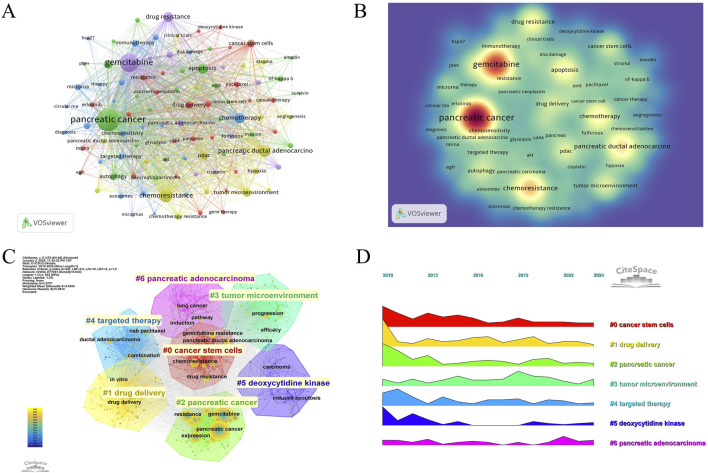
**(A)** Keywords co-occurrence frequency diagram. **(B)** Density map of keywords. **(C)** Clustering map of keywords. **(D)** Peak map of keyword clustering.

The keyword co-occurrence and density visualization were generated using VOSviewer, where keywords were treated as nodes, and their occurrence frequencies were systematically quantified (Figures 5A, B) (Table 6). An increased node size and density in the visualization signifies a higher occurrence rate of keywords in the scrutinized literature. The most frequently occurring keywords included pancreatic cancer (n = 1,213), gemcitabine (n = 685), chemoresistance (n = 290), pancreatic ductal adenocarcinoma (n = 219), and chemotherapy (n = 153), aligning closely with the overarching theme of this study. A co-occurrence network was constructed for 105 keywords with frequencies exceeding 10, revealing stronger inter-keyword associations and clearer co-occurrence relationships, which demonstrated a potential distribution characterized by “supercore, multicenter, and pan-thematic” structures. Additionally, clustering visualizations and peak trend maps were developed from the keyword frequency network to investigate the lateral distributions and evolutionary trends of research hotspots across diverse topics (Figures 5C, D). Seven primary clusters emerged from this study, with labels such as “#0 cancer stem cells,” “#1 drug delivery,” “#2 pancreatic cancer,” “#3 tumor microenvironment,” “#4 targeted therapy,” “#5 deoxycytidine kinase,” and “#6 pancreatic adenocarcinoma,” illustrating the diverse avenues of international research on GEM resistance in PC. It was observed that “tumor microenvironment” and “pancreatic adenocarcinoma” exhibited fluctuations in prominence within the peaks and valleys graph over recent years, emerging as prominent focal points of contemporary research.

**TABLE 6:** High-frequency keywords (top 20).

Rank	Label	Occurrences
1	Pancreatic cancer	1,213
2	Gemcitabine	685
3	Chemoresistance	290
4	Pancreatic ductal adenocarcinoma	219
5	Chemotherapy	153
6	Apoptosis	139
7	Drug resistance	131
8	Gemcitabine resistance	88
9	Tumor microenvironment	82
10	Pdac	80
11	Autophagy	68
12	Cancer stem cells	68
13	Drug delivery	63
14	Immunotherapy	52
15	Prognosis	50
16	Chemosensitivity	43
17	Cancer	41
18	Pancreatic adenocarcinoma	41
19	Combination therapy	40
20	Microrna	40

### 3.6 Literature co-citation analysis

**FIGURE 6:**
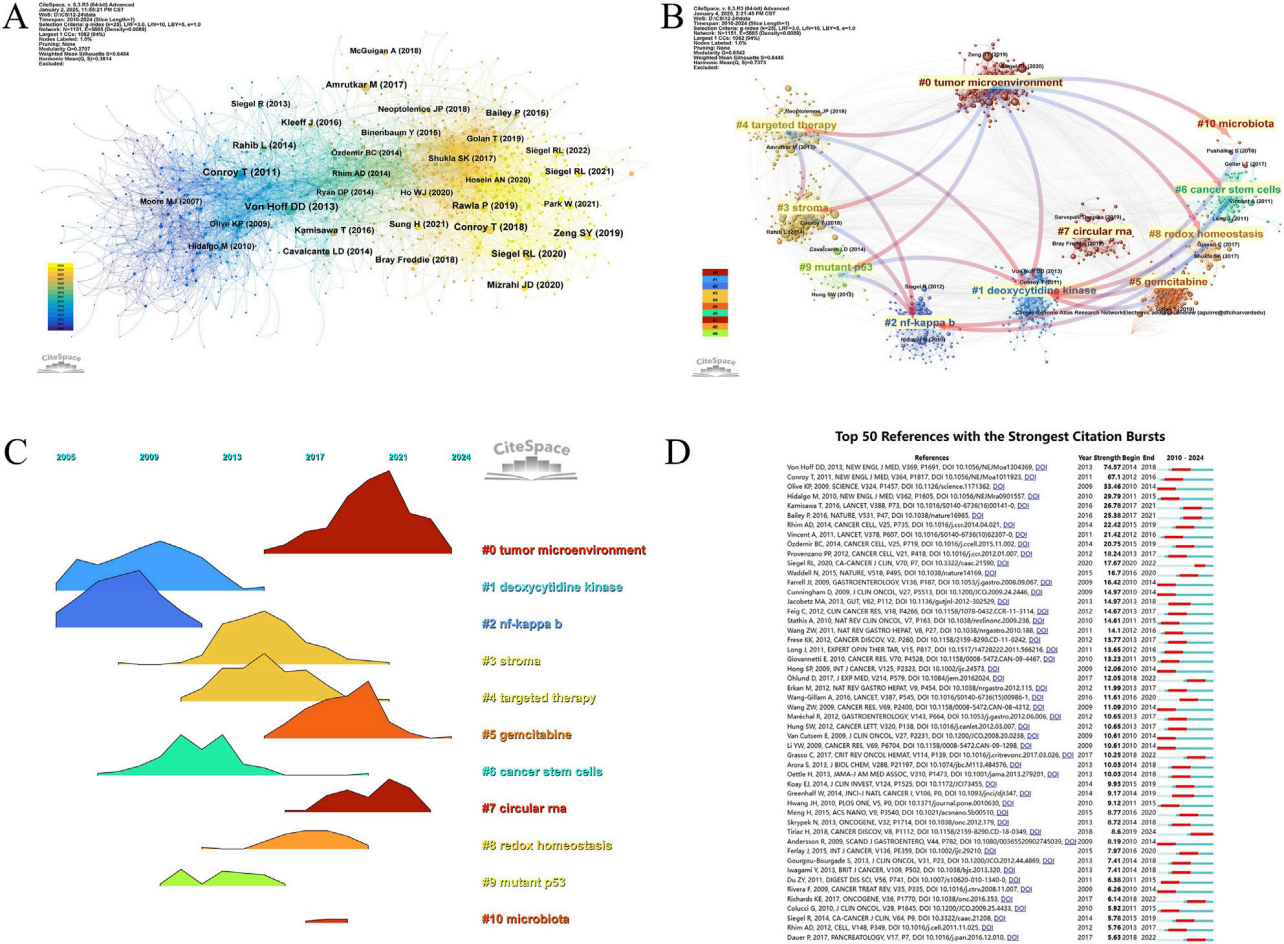
**(A)** Co-cited network of literature. **(B)** Clustering of co-cited literature. **(C)** Peak map of co-cited literature. **(D)** Bursting list of cited literature.

This research utilized a combined citation analysis of the literary network through CiteSpace to pinpoint influential publications and reveal prevalent trends within these studies (Figure 6A; Table 7). Articles such as Conroy T et al.’s “FOLFIRINOX versus gemcitabine for metastatic pancreatic cancer” (New Engl J Med, n = 573), Burris HA et al.’s “Improvements in survival and clinical benefit with gemcitabine as first-line therapy for advanced pancreatic cancer: a randomized trial” (J Clin Oncol, n = 548), and Von Hoff DD et al.’s “Increased survival in pancreatic cancer with nab-paclitaxel plus gemcitabine” (New Engl J Med, n = 521) were among the most frequently cited publications in the network. This highlights the industry’s broad recognition of these high-quality studies. By constructing literature clustering and peak mapping derived from the co-citation network, we aimed to visually elucidate the foundational research base and cutting-edge topics (Figures 6B, C). The mapping results revealed that the “tumor microenvironment” served as the foundational starting point for all clustered themes, further extending into topics such as “deoxycytidine kinase,” “stroma,” “targeted therapy,” “cancer stem cells,” “redox homeostasis,” “microbiota,” and related emerging directions. Notably, cluster #1, focusing on deoxycytidine kinase, explored intersections among these research trajectories. Over the past 15 years, research has primarily centered on deoxycytidine kinase (Cluster 1) and NF-kappa B (Cluster 2). Gradually, emerging topics such as stroma (Cluster 3), targeted therapy (Cluster 4), cancer stem cells (Cluster 6), and mutant p53 (Cluster 9) have gained prominence. The tumor microenvironment (Cluster 0), targeted therapy (Cluster 5), and circular RNA (Cluster 6) have emerged as focal research hotspots, drawing significant scholarly attention in recent years. Furthermore, we analyzed the citation burst periods of key references (Figure 6D). Notably, two articles from the New England Journal of Medicine, authored by Von Hoff DD et al. and Conroy T et al., exhibited the highest burst intensities (n = 74.54, n = 67.1), sustaining attention and citations between 2012 and 2018, thereby driving subsequent research advancements.

**TABLE 7:** Highly co-cited literature (top 10).

Rank	Title	Journal	Author	Citations
1	FOLFIRINOX versus gemcitabine for metastatic pancreatic cancer	New Engl J Med	Conroy T, et al.	573
2	Improvements in survival and clinical benefit with gemcitabine as first-line therapy for patients with advanced pancreas cancer: a randomized trial	J Clin Oncol	Burris HA, et al.	548
3	Increased survival in pancreatic cancer with nab-paclitaxel plus gemcitabine	New Engl J Med	Von Hoff DD, et al.	521
4	Erlotinib plus gemcitabine compared with gemcitabine alone in patients with advanced pancreatic cancer: a phase III trial of the National Cancer Institute of Canada Clinical Trials Group	J Clin Oncol	Moore MJ, et al.	308
5	Projecting cancer incidence and deaths to 2030: the unexpected burden of thyroid, liver, and pancreas cancers in the United States	Cancer Res	Rahib L, et al.	307
6	Inhibition of Hedgehog signaling enhances delivery of chemotherapy in a mouse model of pancreatic cancer	Science	Olive KP, et al.	265
7	Cancer statistics, 2007	CA-Cancer J Clin	Jemal A, et al.	237
8	Gemcitabine resistance in pancreatic ductal adenocarcinoma	Drug Resist Update	Binenbaum Y, et al.	174
9	Pancreatic cancer	Nat Rev Dis Primers	Kleeff J, et al.	162
10	FOLFIRINOX or gemcitabine as adjuvant therapy for pancreatic cancer	New Engl J Med	Conroy T, et al.	158

## 4 Discussion

This study incorporated 2,689 publications on GEM and PC drug resistance research from the WOS database. Analyses involving bibliometry and visualization were performed using CiteSpace and VOSviewer to delve into scholarly work on GEM and PC drug resistance. This research primarily sought to clarify the traits and key areas of heightened attention in the worldwide context of GEM and PC drug resistance. Additionally, this study aimed to predict emerging trends that could provide valuable insights for researchers in GEM and PC drug resistance.

GEM, a chemotherapeutic agent developed by Lilly in 1983, was subsequently demonstrated by Hertel LW of Lilly Research Laboratories in 1990 to exhibit efficacy against a broad spectrum of malignant tumors, establishing it as a preferred therapeutic option in oncology (Hertel et al., 1990); In 1997, Burris of the Cancer Treatment Center of the United States, in a comparative study of GEM and the traditional chemotherapeutic agent 5-fluorouracil (5-Fu) for the treatment of PC, reported that GEM markedly enhanced both the clinical benefit rate and median survival when compared to 5-Fu (Burris and Storniolo, 1997). Due to the established efficacy of GEM in treating advanced PC, the U.S. Food and Drug Administration (FDA) formally approved it as a first-line chemotherapeutic agent for PC. Although GEM demonstrates efficacy in advanced metastatic patients, clinical outcomes remain suboptimal, largely due to the rapid onset of drug resistance, which emerges in the majority of patients within weeks of initiating treatment, significantly impairing prognosis (Avan et al., 2013a). In response to the challenge of drug resistance and the need to enhance chemotherapy responsiveness, there has been a notable increase in studies focused on GEM and PC resistance over the past three decades. Nevertheless, to date, no bibliometric analysis has been conducted in this domain. Firstly, our study results, along with the observed publication trends, demonstrate a dramatic increase in the annual publication output, rising from 74 publications in 2010 to 261 in 2024. This trend underscores the growing research interest in GEM and PC drug resistance over the past 15 years. Despite a modest decline in global publication output from 2022 to 2024, the overall research momentum on GEM and PC drug resistance is expected to continue rising annually (R^2^ = 0.9634).

Furthermore, a thorough examination of the top influential nations, institutions, writers, and periodicals in this field was carried out. Our findings show that China and the United States top the charts in publication numbers, accounting for 35.85% and 32.17% of total publications, respectively. Nonetheless, the United States ranks just above China in total article publications, boasting nearly 15,000 more citations than China, and the mean article citations are about half compared to the United States. This suggests that, despite China’s substantial publication output, its influence in the field remains limited, as the articles lack sufficient persuasive power and require substantial improvement in quality. With regard to institutions, universities from the United States and China dominate the global rankings, with Fudan University leading the world in the number of publications within this field. Based on current trends, cross-regional and inter-institutional collaboration appears to be a key driver in advancing the in-depth exploration of specific research areas. Journal analysis reveals that the top 10 journals collectively published 21.5% of the articles. Notably, in terms of journal citations, *Cancer Research* (n = 414, IF = 12.5, Q1), *Clinical Cancer Research* (n = 1768, IF = 10.4, Q1), and *Journal of Clinical Oncology* (n = 1,434, IF = 42.1, Q1) ranked as the top three journals, each with an impact factor exceeding 10. The results reveal a significant involvement of several top-tier, influential publications in enhancing GEM & PC drug resistance research, highlighting their crucial contribution. In terms of author contributions, Elisa Giovannetti (n = 38) and Xianjun Yu (n = 27) are the leading authors with the highest publication counts. Elisa Giovannetti’s pivotal studies include a landmark 2006 investigation demonstrating the potential of pharmacogenetic approaches to elucidate the mechanisms underlying resistance to GEM and PC. This study elucidated the role of human equilibrium nucleoside transporter protein-1 as a prognostic marker in the treatment of PC patients with GEM (Giovannetti et al., 2006); Furthermore, Crizotinib was shown to specifically target a subpopulation of PC cells, inhibiting cell proliferation, inducing apoptosis, reducing migration, and synergistically interacting with GEM to mitigate the development of drug resistance in PC patients (Avan et al., 2013b); Xianjun Yu, a prominent researcher at the Institute of Pancreatic Tumor Research at Fudan University, has extensively investigated the resistance mechanisms of PC cells to GEM. The Institute of Pancreatic Tumor Research at Fudan University has consistently ranked among the top institutions in terms of scientific publications in this field. Notably, one of Yu’s studies identified glutathione peroxidase-1 (GPx1) as a key regulator of epithelial-mesenchymal transition (EMT) and chemoresistance through modulation of the AKT/GSK3β/Snail signaling axis in PC, suggesting that GPx1 may serve as a potential predictive biomarker for GEM treatment in PC patients (Meng et al., 2018). hNF4α has been identified as a prognostic marker for overall survival in PC, essential for cell proliferation, and contributes to GEM resistance by down-regulating hENT1 (Sun et al., 2019). In contrast, our findings indicate that David A. Tuveson is the most cited author in this field, with several of his significant studies published in top-tier journals such as *Cancer Research*, *Cell*, and *Science*, aligning with our own results.

## 5 Hot spots and frontiers

This research concentrates on identifying key areas and boundaries in GEM & PC drug resistance studies. Our examination of key terms cited sources, and their respective outbreaks revealed that GEM & PC drug resistance studies primarily concentrate on these domains:

(1) The role of the tumor microenvironment in the development of GEM resistance in PC is crucial. This microenvironment is characterized by a dense extracellular matrix, an abundance of fibroblasts, a disorganized capillary network, and a heterogeneous population of immune cells. The non-cancerous elements found in PC play a substantial role in creating varied cell types and fostering drug resistance, which assists in the growth and spread of tumor cells (Halbrook et al., 2023). The extracellular matrix (ECM) constitutes a fundamental component of the tumor microenvironment in PC, functioning as a formidable physical barrier. It is widely recognized that in PC, activated cancer-associated fibroblasts (CAFs) generate a dense fibrotic network by secreting substantial amounts of collagen, hyaluronic acid, and fibronectin, thereby markedly elevating interstitial fluid pressure (IFP) and hindering the penetration of small-molecule chemotherapeutics, such as GEM, into the tumor core (Chen X. et al., 2022; Geng et al., 2022). Consequently, this impedes both the delivery and diffusion of chemotherapeutic agents in PC, thereby attenuating their cytotoxic efficacy against PC cells (Hosein et al., 2020). Hyaluronic acid and type I collagen are pivotal in this process, with the former obstructing the effective delivery of GEM by elevating intratumoral pressure and inducing vascular collapse (DuFort et al., 2016), while the latter counteracts GEM efficacy by establishing a robust physical barrier and upregulating drug resistance-associated proteins (Gu et al., 2021). Clinical strategies have been explored to enhance drug delivery efficiency by employing hyaluronidase (e.g., PEGPH20) to degrade hyaluronic acid in combination with GEM (Hingorani et al., 2016). Recent studies have demonstrated that the hypoxic tumor microenvironment plays a crucial role in driving GEM resistance in PC cells via multiple mechanisms. For instance, hypoxia-inducible factor-1α (HIF-1α) activation stimulates drug efflux pumps while suppressing inflammation-associated pathways, subsequently upregulating multidrug resistance genes (e.g., MDR1/ABCB1), enhancing the expression of efflux transporters such as P-glycoprotein (P-gp) (Chen S. Y. et al., 2022), expediting GEM efflux from cells, and thereby diminishing its intracellular bioavailability. Thereby further diminishing the therapeutic efficacy of GEM (Thomas and Tampé, 2020; Wang et al., 2014). Literature reports indicate that HIF-1α confers resistance to GEM-induced DNA synthesis inhibition by augmenting glycolysis (e.g., upregulation of GLUT1 and LDHA), thereby promoting tumor cell reliance on anaerobic metabolism for survival (Dai et al., 2020). Moreover, hypoxic conditions further attenuate GEM efficacy by upregulating metabolic pathways, including glycolysis and pyrimidine biosynthesis (Xin et al., 2020). Hypoxia further enhances the self-renewal capacity of cancer stem cells (CSCs) by activating the Notch and Wnt/β-catenin signaling pathways. CSCs exhibit high expression levels of ABC transporters and anti-apoptotic proteins (e.g., Bcl-2), rendering them intrinsically resistant to chemotherapeutic agents such as GEM (Kallifatidis et al., 2011). The infiltration of immune cells and the immunosuppressive milieu within PC are prominent features that not only foster resistance to immune checkpoint blockade therapies but also enhance tumor growth, invasion, and metastasis. Various immune cell groups, such as tumor-linked macrophages, myeloid-originated suppressors, and cancer-penetrating lymphocytes, play a vital role in this mechanism and influence the responsiveness of PC cells to GEM (Kajiwara et al., 2023; Mitchem et al., 2013); The processes through which the tumor microenvironment influences GEM resistance in PC are complex and perpetually shifting. Beyond the aforementioned known factors, several challenges remain, including the heterogeneous sensitivity of distinct tumor cell populations to GEM, which leads to suboptimal therapeutic outcomes. Furthermore, the complex interplay between signaling pathways in the tumor microenvironment and their role in drug resistance is yet to be fully comprehended. Future investigations should aim to systematically unravel the complex interactions of these signaling pathways through integrated multi-omics approaches, thereby identifying novel therapeutic targets.
(2) Progress of targeted therapy in the study of GEM and PC resistance: Targeted therapy, recognized as a promising therapeutic approach, specifically targets relevant molecular sites to inhibit tumor growth, invasion, and metastasis. The approach has attracted notable focus in the study of PC, especially regarding GEM resistance, where researchers are examining diverse anti-angiogenic agents. Notably, Erlotinib’s action on the epidermal growth factor receptor (EGFR) has been effective in extending both general and survival without disease progression, especially in individuals with locally advanced or metastatic PC. Significantly, this dual therapy markedly enhances the one-year survival rate, indicating a viable method to reduce drug resistance (Månsson et al., 1985); Bevacizumab, recognized as the pioneering anti-angiogenic monoclonal antibody, distinctively attaches to vascular endothelial growth factor (VEGF), thus hindering neovascularization and impeding tumor advancement. This is being explored as a treatment alternative to combat GEM resistance in advanced PC patients (Watkins et al., 2014). The latest research has pinpointed insulin-like growth factor 1 (IGF-1) as a cytokine pivotal in controlling key cell functions like proliferation, movement, and survival. IGF-1 impacts these processes through its attachment to IGF-1R, activating crucial subsequent signaling routes like MAPK and PI3K/AKT. PC lesions display increased levels of IGF-1 and its receptor IGF-1R, markedly surpassing what is seen in healthy tissues. This overexpression correlates with adverse pathological features and poor prognosis (Wan et al., 2019). Furthermore, preclinical models have demonstrated that blocking the interaction between IGF-1 and its receptor enhances the sensitivity of the PANCI PC cell line to GEM (Mutgan et al., 2018). Ganitumab, a monoclonal antibody targeting IGF-1R, prevents IGF-1 from binding to its receptor. Clinical trials have demonstrated that Ganitumab, in combination with GEM, significantly enhances therapeutic efficacy and reduces the emergence of drug resistance, compared to GEM monotherapy, in the treatment of metastatic PC (Fuchs et al., 2015). Despite these advances, several challenges remain in the development of targeted therapies for PC, including insufficient discovery of novel targets, limited therapeutic benefit observed in animal models, and a lack of successful translation from basic research to clinical practice. Moreover, issues such as drug resistance and the toxic side effects of targeted agents urgently require resolution.
(3) Circular RNA (circRNA) has emerged as a prominent focus of research concerning GEM resistance in PC: In recent years, circRNA has garnered significant attention as a critical factor in the pathogenesis of PC and its resistance to GEM. High-throughput sequencing analyses (Li J. et al., 2018; Li et al., 2018 Z.) have revealed that a substantial number of circRNAs exhibit aberrant expression in PC tissues and cell lines, suggesting a pivotal role in the epigenetic regulation of tumor progression; For instance, Xu et al. (2018) identified several circRNAs, including circ-101672 and circ-102747, which are aberrantly expressed in GEM-resistant PC cells. These findings led to the hypothesis that circ-101672 and circ-102747 may interact with drug-resistant miRNAs, thereby contributing to the mechanisms underlying GEM resistance; Shao et al. (2018) demonstrated that two circRNAs (chr4: 52729603-52780244+, chr14: 101402109-101464448+) are strongly associated with GEM resistance. They hypothesized that these circRNAs could interact with miR-124-3p and miR-145, thereby modulating the drug resistance pathways; Another study reported that circ-0005785 harbors a binding site for miR-181b (Li et al., 2016), which suppresses deubiquitinating enzyme activity and potentiates the drug resistance mechanisms. Further research indicated that circ-0005785 (Takiuchi et al., 2013) contains a binding site for miR-181b, which not only inhibits deubiquitinating enzyme activity but also activates the nuclear factor-κB signaling pathway, thereby contributing to GEM resistance in PC cells; Consequently, it was hypothesized that circ-0005785 might facilitate GEM resistance in PC cells through the sequestration of miR-330-5p; Additionally, circHIPK3 was found to be aberrantly overexpressed in PC tissues, where it enhances RNA resistance mechanisms by sequestering miR-330-5p. This upregulation of RASSF1 contributes to the promotion of GEM resistance in PC cells (Liu et al., 2020). Furthermore, circular RNAs may attenuate the inhibitory effects of GEM on DNA synthesis by augmenting DNA repair capacity or modulating cellular metabolic pathways. Emerging evidence suggests that in PC, circABCC4 facilitates aerobic glycolysis by promoting the nuclear translocation of PKM2 (pyruvate kinase M2) and concurrently enhances non-homologous end-joining (NHEJ) DNA repair. This dual mechanism enables tumor cells to withstand GEM-induced DNA damage, thereby markedly enhancing drug resistance (He et al., 2025). Moreover, cyclic RNAs have been implicated in the indirect modulation of drug transporter protein expression, thereby diminishing the intracellular accumulation of GEM. Although less extensively studied, accumulating evidence indicates that specific cyclic RNAs (e.g., circFADS1) facilitate drug efflux and contribute to chemoresistance by activating the Wnt/β-catenin pathway and potentially upregulating ABC transporter family members (e.g., MDR1/ABCB1) (König et al., 2005; Wang et al., 2021). Similar resistance mechanisms have been observed in response to other chemotherapeutic agents, implying that cyclic RNAs may modulate GEM efficacy through analogous pathways. Collectively, circular RNAs orchestrate GEM resistance via multifaceted regulation of resistance-associated genes, encompassing miRNA sponge activity, enhanced DNA repair, metabolic reprogramming, and drug efflux. Current research has partially elucidated the potential role of circRNAs in PC chemoresistance; however, further validation through clinical sample analysis and *in vivo* experimentation remains imperative. We posit that future investigations should delve deeper into the interplay between circRNAs and other drug resistance mechanisms while developing targeted delivery systems to enhance therapeutic precision. Therefore, specific circRNAs hold promise as potential therapeutic targets for PC.

In summary, the critical challenges and pressing research priorities in this field over the next 5 years include: Future investigations should leverage multi-omics approaches—including genomics, transcriptomics, proteomics, and metabolomics—to comprehensively elucidate the molecular mechanisms underlying PC cell resistance to GEM. Key aspects of resistance include the regulatory influence of non-coding RNAs, epigenetic modifications, TME interactions, and immune evasion. While multi-omics technologies offer a holistic framework for deciphering GEM resistance, their inherent challenges primarily arise from the biological complexity of PC and technological constraints. Key limitations include the difficulty in balancing technical sensitivity and throughput, inefficiencies in clinical translation and functional validation, as well as substantial economic costs. Potential solutions include the establishment of an integrated data analysis platform (e.g., AI-powered multi-omics cloud-based analytical tools), standardization of data formats and quality control protocols, and advancements in single-cell and spatial genomics technologies to enhance resolution and clinical applicability. Additionally, future research should prioritize the development of combination therapeutic strategies targeting drug resistance mechanisms, including KRAS-targeted therapies and DNA damage repair inhibitors (e.g., PARP inhibitors and ATR/CHK1 inhibitors). Emerging technologies, including liquid biopsy and AI-driven analytical methods, should be integrated to advance the implementation of precision medicine in PC therapy. By fostering interdisciplinary collaboration and technological innovation, we can overcome the therapeutic barriers associated with GEM resistance and enhance both the survival outcomes and quality of life of PC patients.

Inevitably, our study has several limitations. Primarily, it relies on bibliometric analysis, which emphasizes publication and citation counts while lacking an in-depth evaluation of study quality. Furthermore, as the data were exclusively sourced from WOSCC, relevant literature from other databases such as Scopus and PubMed—particularly non-English publications—may have been inadvertently excluded, potentially introducing a bias in the findings. Such limitations may contribute to a potential bias in the study’s conclusions. Nevertheless, we contend that this bibliometric analysis provides valuable insights into emerging research hotspots in GEM and PC studies, thereby making a meaningful contribution to the field of literature analysis.

## Data Availability

The original contributions presented in the study are included in the article/supplementary material, further inquiries can be directed to the corresponding author.
